# Early circulating tumour DNA kinetics measured by ultra-deep next-generation sequencing during radical radiotherapy for non-small cell lung cancer: a feasibility study

**DOI:** 10.1186/s13014-020-01583-7

**Published:** 2020-05-29

**Authors:** G. M. Walls, L. McConnell, J. McAleese, P. Murray, T. B. Lynch, K. Savage, G. G. Hanna, D. Gonzalez de Castro

**Affiliations:** 1 Centre for Cancer Research & Cell Biology, Queen’s University Belfast, 97 Lisburn Road, Belfast, BT9 7AE Northern Ireland; 2 Cancer Centre Belfast City Hospital, Belfast Health & Social Care Trust, 51 Lisburn Road, Belfast, BT9 7AB Northern Ireland; 3 Northern Ireland Biobank, Health Sciences Building, Queen’s University Belfast, 97 Lisburn Road, Belfast, BT9 7AE Northern Ireland; 4 Sir Peter MacCallum Department of Oncology, University of Melbourne, 305 Grattan St, Melbourne, VIC 3000 Australia

**Keywords:** ctDNA, Ultra-deep NGS, Radical radiotherapy, Lung cancer

## Abstract

**Background:**

The evaluation of circulating tumour DNA (ctDNA) from clinical blood samples, liquid biopsy, offers several diagnostic advantages compared with traditional tissue biopsy, such as shorter processing time, reduced patient risk and the opportunity to assess tumour heterogeneity. The historically poor sensitivity of ctDNA testing, has restricted its integration into routine clinical practice for non-metastatic disease. The early kinetics of ctDNA during radical radiotherapy for localised NSCLC have not been described with ultra-deep next generation sequencing previously.

**Materials and methods:**

Patients with CT/PET-staged locally advanced, NSCLC prospectively consented to undergo serial venepuncture during the first week of radical radiotherapy alone. All patients received 55Gy in 20 fractions. Plasma samples were processed using the commercially available Roche AVENIO Expanded kit (Roche Sequencing Solutions, Pleasanton, CA, US) which targets 77 genes.

**Results:**

Tumour-specific mutations were found in all patients (1 in 3 patients; 2 in 1 patient, and 3 in 1 patient). The variant allele frequency of these mutations ranged from 0.05–3.35%. In 2 patients there was a transient increase in ctDNA levels at the 72 h timepoint compared to baseline. In all patients there was a non-significant decrease in ctDNA levels at the 7-day timepoint in comparison to baseline (*p* = 0.4627).

**Conclusion:**

This study demonstrates the feasibility of applying ctDNA-optimised NGS protocols through specified time-points in a small homogenous cohort of patients with localised lung cancer treated with radiotherapy. Studies are required to assess ctDNA kinetics as a predictive biomarker in radiotherapy. Priming tumours for liquid biopsy using radiation warrants further exploration.

## Introduction

Circulating tumour DNA (ctDNA) describes tumour-derived DNA fragments released into peripheral blood through necrosis, apoptosis and spontaneous release [1]. The term ‘liquid biopsy’ has been used to describe the evaluation of total cell-free DNA (cfDNA) from clinical blood samples, and compared with traditional tissue biopsy, liquid biopsy can be faster, less invasive and more comprehensive in terms of reflecting tumour heterogeneity. The ctDNA must be identified amongst the cfDNA produced by non-malignant cells from around the body [2]. There are conflicting data for total cell-free circulating DNA trends during treatment, and cfDNA is less useful as a prognostic biomarker [3].

The half-life of ctDNA is estimated to be up to 2 hours, and is dependent on factors including cell turnover, tumour size, excretion in bodily fluids and degradation rate by circulating nucleases [4]. Therefore, in non-metastatic cancer, concentration ranges of ctDNA, considered as fractions of total cell-free DNA, vary between tumour types, ranging from undetectable in prostate cancer [5] to 0.02–3.2% in non-small cell lung cancer (NSCLC) [6]. The historically poor sensitivity of ctDNA testing, has restricted its integration into routine clinical practice in non-metastatic disease [7].

The emerging potential clinical utilities of ctDNA in lung cancer management include screening [8], histological and molecular subtyping [9, 10], disease burden assessment [11], overall prognosis [12, 13], systemic treatment response assessment [14] (oncogenic-driven cases included [15, 16]), identification of resistance mechanisms [17] and response to local consolidative radiotherapy [18].

Although approximately 45% stage I-III NSCLC cases receive radical radiotherapy as multi- or single modality treatment [19], there is only one published series on the impact of radiotherapy on cfDNA in NSCLC [20]. Furthermore, the paucity of data on ctDNA dynamics during radiotherapy across other tumour sites means there are few transferable lessons about any possible interplay in NSCLC [20–26] As the anti-tumour activity of radiotherapy is achieved through DNA damage-mediated cell death, it is expected that an interaction will be observed. Additional evidence for transient ctDNA increases on commencing treatment may support the evaluation of radiotherapy as a preparatory procedure for liquid biopsy. This prospective pilot study aimed to demonstrate the suitability of ultra-deep NGS ctDNA quantitation for examination of the relationship between radiotherapy delivery and ctDNA dynamics in non-metastatic NSCLC.

## Methods and materials

### Patient selection

Patients receiving radical radiotherapy alone for locally advanced, histologically confirmed NSCLC provided consent for serial venepuncture during the first week of treatment. All patients were deemed unsuitable for concurrent chemoradiation. Routine diagnostic investigations included an 18-FDG-PET-CT for all patients, and TNM8 staging was applied [27]. Patients were approached at the radiotherapy consent clinic regarding study participation. Routine clinical assessments were recorded including ECOG PS and smoking history. Response assessments were carried out by a Consultant Radiologist with expertise in lung cancer.

### Radiotherapy

All patients received 55Gy in 20 fractions over 4 weeks planned with the intensity modulated radiotherapy technique and delivered as 6MV arc therapy, with daily online cone beam-CT image guidance, treating Monday to Friday for 4 weeks. All target volumes were subject to peer review [28]. Patients were routinely clinically assessed once per week by an Oncologist or Radiographer.

### Sample Collection & Processing

**Fig. 1:**
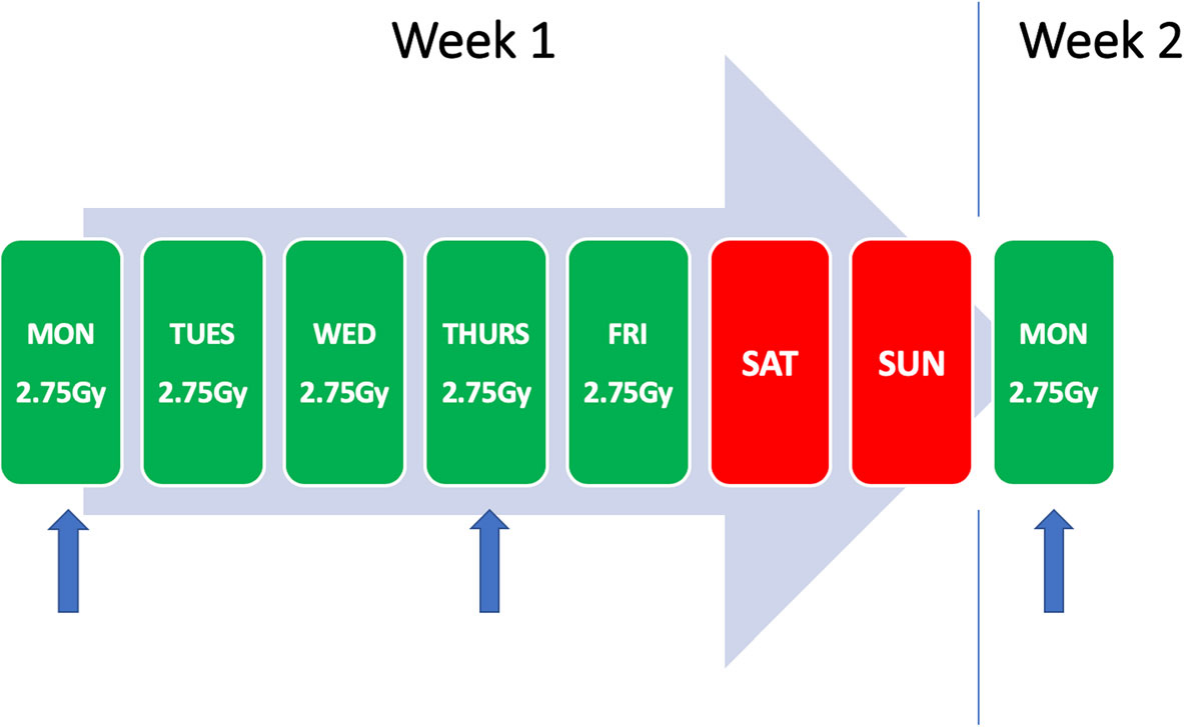
Schema of plasma collection time-points. (blue arrows = venepuncture time-points)

The time, date and location of blood draws were agreed with each patient in consideration with their radiotherapy appointments and no additional hospital visits were required as part of the study. Patients provided 20 mL of blood at 3 different time periods i) immediately prior to fraction 1, ii) 72 h after fraction 1 and iii) 7 days after fraction 1, as illustrated in the study schema in Fig. 1. Samples were transferred to the local Biobank where all blood samples were processed within a 2-h time period. Each blood sample was initially collected via a vacutainer system into 2 × 10 mL EDTA tubes and transported in ambient temperature to the dedicated Biobank laboratory. The EDTA tubes were centrifuged at 2000 g for 10 min producing 10 mL of plasma and a 1 mL buffy coat sample (which was not available for processing). The plasma was then decanted into a 15 mL conical tube and centrifuged again at 2000 g for 10 min to produce cell free plasma and frozen at − 80 °C.

### Library Generation & Next-Generation Sequencing

Plasma samples were processed using the commercially available Roche AVENIO Expanded kit (Roche Sequencing Solutions, Pleasanton, CA, US) as per the manufacturer’s protocol. Briefly, DNA was extracted using the AVENIO cfDNA Isolation Kit. Libraries were prepared, hybridised and analysed according to the AVENIO ctDNA Analysis Kits Reagent Workflow User Guide (Version 1.1; Software Version 1.0.0). Following extraction, DNA was washed using the AVENIO cfDNA Isolation Kit and quantified using the Qubit High Sensitivity assay kit and Qubit 2.0 Fluorometer (Thermo Fisher Scientific, Massachusetts, USA). In order to assess average fragment size, the 4200 TapeStation System using the D1000 ScreenTape with D1000 Reagents (Agilent Technologies, California, USA) was used. Library preparation involved an adapter ligation, a bead clean up, PCR amplification and final bead clean up step and incubated overnight with the AVENIO ctDNA Expanded Panel (Roche Sequencing Solutions, Pleasanton, CA, US). Hybridised libraries underwent a streptavidin bead clean up, PCR amplification and a final bead clean up step before DNA quantification. The pooled libraries were diluted to 4 nM, sequenced on the Illumina NextSeq 500 (Illumina Inc., San Diego, CA, US) and data was analysed using Roche AVENIO ctDNA Analysis Software Version 1.0.0 (Roche Sequencing Solutions, Pleasanton, CA, US). This software includes bioinformatics methods from CAPP-Seq and integrated digital error suppression providing analytical sensitivity and specificity of > 99% at allele frequencies down to 0.5–1% [29]. Roche AVENIO Expanded panel targets 77 genes including single nucleotide variants (SNVs), indels, fusion genes and copy number variants (CNVs) [30]. Between 4.3 and 5 mL of plasma were used to extract cfDNA and an average of 30 ng (range 13–42 ng) of cfDNA was extracted. Sequencing yielded an average of 49,528,620 reads per sample during 300 cycles (range 41,813,828-54,599,643) and the mean unique sequencing depth was 5054x ± 1098 SD.

### Statistical analysis

Statistical significance of ctDNA levels between timepoints was calculated using the non-parametric Friedman test. Whilst day 3 and day 7 measurements follow normal distribution (parametric data), baseline measurements do not (non-parametric data).

### Ethics & Governance

Samples in this study were acquired from the local Biobank who have ethical approval (REC reference 16/NI/0030) for the collection and release of human tissue samples and de-identified data from consented patients [31].

## Results

### Clinical characteristics

Five patients aged 63–85 with histologically confirmed NSCLC stage II-III disease were enrolled onto this study between February and June 2019. Standard molecular analysis for our centre was complete in 1 of 3 applicable patients, in a case of adenocarcinoma (Table 1). All patients completed radiotherapy without experiencing grade 3 or above toxicity.

**Table 1:** Clinical characteristics of patient cohort

Patient	Age	ECOG PS	Co-morbidities	Tobacco	T-stage	N-stage	M-stage	Histology	EGFR	ALK	PD-L1
1	82	1	sciatica	Ex (60 PYH)	2a	2	0	adenocarcinoma	unk	unk	unk
2	68	1	TIA, hypothyroidism, dyslipidaemia, MS	Ex (unk)	2a	1	0	squamous cell	N/A	N/A	1–49%
3	83	1	macular degeneration, BPH, dyslipidaemia, HTN	Ex (unk)	4	2	0	adenosquamous	unk	unk	unk
4	61	2	MI, Raynaud’s	Active	3	2	0	squamous cell	N/A	N/A	< 1%
5	69	0	N/A	Ex (unk)	3	1	0	adenocarcinoma	WT	WT	> 50%

(*ECOG PS* Eastern Cooperative Oncology Group Performance Status, *PYH* pack year history, *unk* unknown, *WT* wild-type, *TIA* transient ischaemic attack, *MS* multiple sclerosis, *HTN* hypertension, *BPH* benign prostatic hyperplasia, *MI* myocardial infarction, *N/A* not applicable)

### ctDNA analysis

**Fig. 2:**
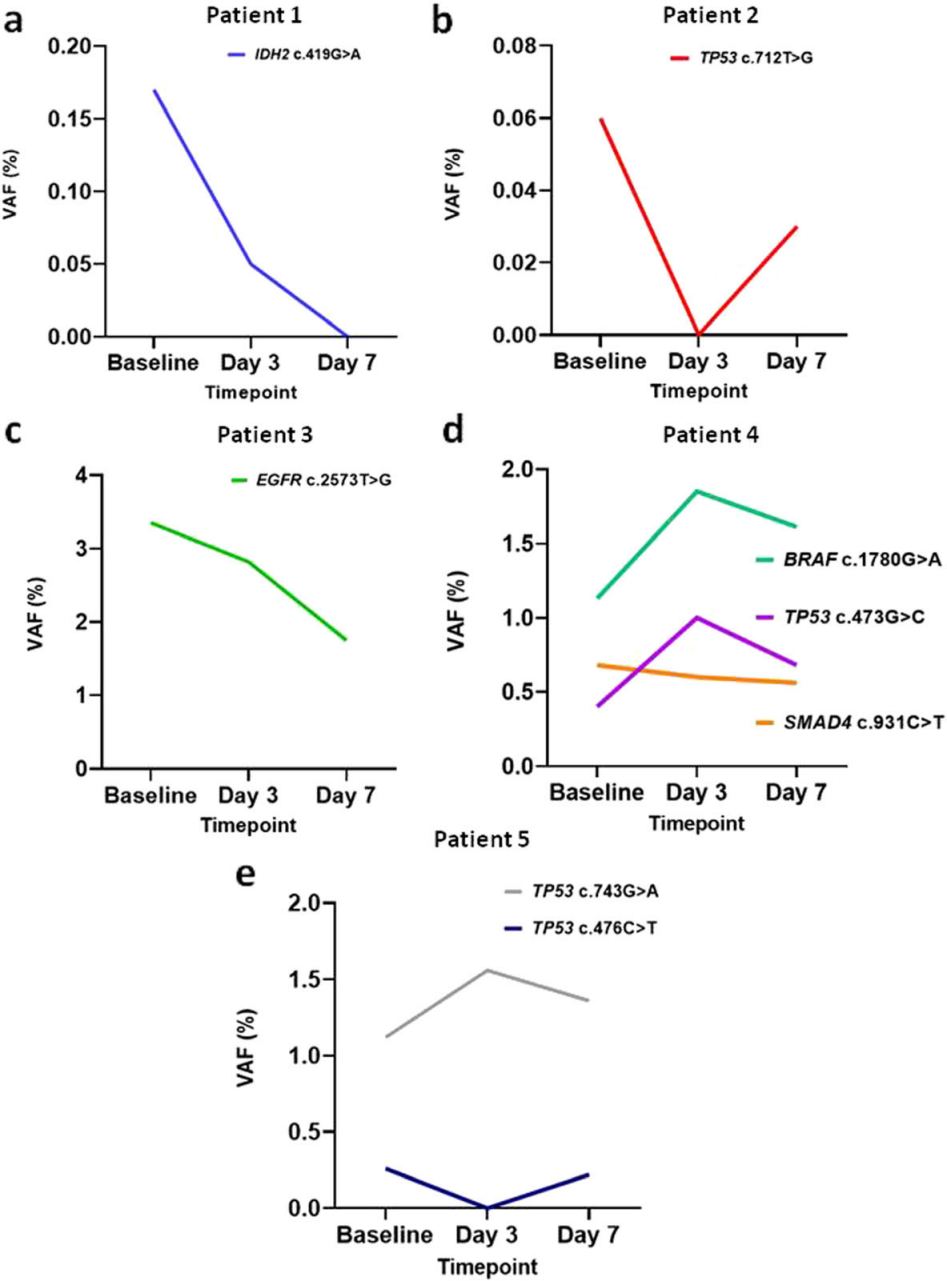
Line graphs depicting the VAF of mutations (labelled on each graph) detected in patients 1 to 5 (a-e, respectively) at baseline, 3 days and 7 days mid-treatment

The median time from sample collection to processing was 25 mins (10–45), and the median time from processing to freezing was 30 mins (20–50). Potential tumour-specific mutations were found in all patients at baseline (1 in 3 patients; 2 in 1 patient, and 3 in 1 patient) and then tracked 3 days and 7 days post radiation as detailed in Table 2. The variant allele frequency (VAF) of these mutations ranged from 0.05–3.35%, consistent with somatic mutations originating in ctDNA. Mutations detectable in plasma were decreased at 7 days in all patients. In 2 patients there was a transient increase in ctDNA levels at the 72 h timepoint compared to baseline (Fig. 2). Mean ctDNA levels for all patients show a slight increase at 72 h and a decrease at day 7 in comparison to the baseline measurement. This numerical difference is not significant, *p* = 0.4627, based on a one-way ANOVA test.

**Table 2:** amount of DNA assessed, coding and amino acid changes, sequencing depths and VAFs for each sample

Sample ID	Isolated DNA Mass (ng)	Gene	Coding Change	Amino Acid Change	Allele Fraction	No. Mutant Molecules per mL	Unique depth
Patient 1-day 1	22.49	*IDH2*	c.419G > A	p.Arg140Gln	0.17%	2.6	4506
Patient 1-day 3	23.21	*IDH2*	c.419G > A	p.Arg140Gln	0.05%	0.783	4622
Patient 2-day 1	38.61	*TP53*	c.772G > T	p.Glu258*	0.06%	1.67	6361
Patient 2-day 3	35.69	Variant ND	N/A	N/A	N/A	N/A	5625
Patient 2-day 7	37.83	Variant ND	N/A	N/A	N/A	N/A	5334
Patient 3-day 1	30.16	*EGFR*	c.2573 T > G	p.Leu858Arg	3.35%	66.7	4579
Patient 3-day 3	39.65	*EGFR*	c.2573 T > G	p.Leu858Arg	2.82%	73.7	4911
Patient 3-day 7	40.37	*EGFR*	c.2573 T > G	p.Leu858Arg	1.75%	51.9	6116
Patient 4-day 1	26.33	*TP53*	c.473G > C	p.Arg158Pro	0.40%	6.98	5630
Patient 4-day 1	26.33	*SMAD4*	c.931C > T	p.Gln311*	0.68%	11.8	5630
Patient 4-day 1	26.33	*BRAF*	c.1780G > A	p.Asp594Asn	1.13%	19.7	5630
Patient 4-day 3	43.42	*TP53*	c.473G > C	p.Arg158Pro	1.00%	28.7	6782
Patient 4-day 3	43.42	*TP53*	c.434 T > C	p.Leu145Pro	0.11%	3.16	6782
Patient 4-day 3	43.42	*SMAD4*	c.931C > T	p.Gln311*	0.60%	17.2	6782
Patient 4-day 3	43.42	*BRAF*	c.1780G > A	p.Asp594Asn	1.85%	53.2	6782
Patient 4-day 7	32.89	*TP53*	c.473G > C	p.Arg158Pro	0.68%	17.1	5662
Patient 4-day 7	32.89	*SMAD4*	c.931C > T	p.Gln311*	0.56%	14.1	5662
Patient 4-day 7	32.89	*BRAF*	c.1780G > A	p.Asp594Asn	1.61%	40.7	5662
Patient 5-day 1	17.94	*TP53*	c.743G > A	p.Arg248Gln	1.12%	13.3	3806
Patient 5-day 1	17.94	*TP53*	c.476C > T	p.Ala159Val	0.26%	3.05	3806
Patient 5-day 3	13.13	*TP53*	c.743G > A	p.Arg248Gln	1.56%	13.5	2430
Patient 5-day 7	33.09	*TP53*	c.743G > A	p.Arg248Gln	1.36%	29.7	5143
Patient 5-day 7	33.09	*TP53*	c.476C > T	p.Ala159Val	0.22%	4.81	5143

(*ND Not detected, *N/A* Not applicable)

### Clinical outcomes

At 3 months post-treatment 4 of 5 patients were alive (Table 3). Patient 3’s tumour exhibited the least response to radiotherapy as assessed by changes in volume and the patient expired from disseminated relapse at 3 months following treatment, without accessing EGFR-targeted therapy due to poor fitness. According to volumetric assessment of the primary and nodes at 3 months confirmed a good response in all other patients. All patients had a decrease in primary tumour volume and the mean decrease was − 60% (− 8% to − 95%). Patient 4 had an increase in some nodes after radiotherapy but appearances were in keeping with cystic change (21 Hounsfield Units), and therefore a good treatment response. In Table 3, if more than one node was FDG-avid, the largest node’s size was recorded.

**Table 3:** Clinical outcomes of study participants

Patient	6-Month ECOG PS	Baseline Volumes (cm^**3**^)		Volumes at 3 Months (cm^**3**^)		Net Change in Primary (%)
		Primary	Node	Primary	Node	
1	unk	36.1	0.10	17.2	0.01	−52%
2	unk	8.2	0.06	3.1	0.00	−62%
3	5	187.4	0.27	172.0	0.27	−8%
4	unk	33.5	2.14	1.8	2.57	−95%
5	0	22.4	1.43	3.6	0.07	− 84%

## Discussion

In recent years, liquid biopsy has obtained a place in standard clinical care for its role in selection of patients for third generation tyrosine kinase inhibitors in *EGFR*-mutant NSCLC after progression on first/second generation treatment, by detection of the p.T790M mutation [32]. The potential for ctDNA analysis as a predictive or prognostic biomarker continues to expand in lung cancer, but most of the clinical utility of ctDNA remains unvalidated [33].

Compared to needle tissue biopsy, liquid biopsy results in reduced risk to the patient and reduced processing time [34]. Poor sensitivity of ctDNA testing methodologies such as allele-specific PCR restricted uptake of this technique previously, however modern next-generation sequencing (NGS) can overcome this issue with sensitivities in excess of 90% reported [6].

In academic studies of metastatic lung cancer, ctDNA levels have been shown to decrease during treatment with cytotoxic [14] and targeted agents [15]. Trough values during systemic therapy have been correlated with disease-free interval and raised levels off-treatment appear to pre-empt clinical relapse by 5.2 months [35].

Similarly, following surgery ctDNA levels are known to plummet within 1 day [36]. Levels immediately post-surgery can indicate minimal residual disease detection, which may guide the clinical management [37]. Radiological relapse post-surgery has been predicted by ctDNA increases up to 5.2 months prior [38]. However, multiple studies have also shown that patients with undetectable ctDNA can develop relapse [35].

There is only one known published report of cfDNA monitoring during radiotherapy for lung cancer [20]. In this study of 17 patients, cfDNA was assessed after each quarter of the radiotherapy treatment course with digital PCR, and NGS where possible (2 cases of thoracic radiotherapy; 2 cases of cranial stereotactic radiosurgery). A net decrease in cfDNA was observed in most cases after radiotherapy, with a modest increase observed during the course of radiotherapy. The 2 patients receiving thoracic radiotherapy who had ctDNA analysis completed by NGS had no detectable ctDNA prior to treatment. After commencing treatment ctDNA became detectable and rose during the first week, followed by a subsequent decrease. This study had a heterogenous population of radiotherapy treatments for early stage primary tumours and brain metastases, and time-points for plasma sampling varied considerably. Thoracic radiotherapy was planned with a 3D-conformal technique, brain radiotherapy was completed with a stereotactic approach.

Here we report a small, prospective, observational pilot study of patients with lung cancer, where the feasibility of applying NGS ctDNA analysis in non-metastatic NSCLC patients receiving radiotherapy was demonstrated. The detected mutations were identified at baseline and subsequent time-points during radiotherapy for all cases, with values ranging from 0.06 to 3.35%. All patients in this feasibility study were found to have reduced ctDNA (ranging from 0 to 1.75%) at 7 days from commencing radiotherapy (5 fractions delivered), although the difference was not statistically significant. This is in keeping with an absolute reduction in viable tumour cells available to release DNA, or impaired DNA release processes in remaining tumour cells. Other possibilities include cellular senescence in response to sub-lethal DNA damage, and differential lethality in tumour clones prone to DNA secretion.

In keeping with this feasibility analysis, unpublished data (*n* = 55) presented at the ASTRO Conference 2017 examining ctDNA in stage I-III NSCLC managed with surgery, radiotherapy and chemoradiation demonstrated a decrease with treatment generally [26]. Mid-treatment and post-treatment ctDNA levels during (chemo-)radiation correlated with progression-free and overall survival. In another unpublished NSCLC cohort, approx. 40% (5/12) patients demonstrated elevated ctDNA early in the course of stereotactic radiotherapy [25].

The pilot findings are also in keeping with evaluations of head and neck cancer patients, where viral-associated DNA, considered ctDNA, appeared to decrease during the course of radical radiotherapy [23, 24]. Furthermore, subsets of patients (approx. 20%) in both studies exhibited a transient rise prior to the eventual decrease (3/14 [23] and 2/10 [24]). In addition, transient rises in ctDNA have been observed following systemic therapy, such as immunotherapy in melanoma [39], neoadjuvant combined cytotoxic/biologic combination therapy in breast carcinoma [40] and tyrosine kinase inhibition in *EGFR*-mutant NSCLC [41].

This pilot study demonstrates the feasibility of applying ctDNA-optimised NGS protocols through specified time-points in a small, homogenous cohort of patients treated with modern radiotherapy planning for locally advanced NSCLC. However, as the kinetics of ctDNA during radical radiotherapy have not been hitherto described in relation to lung cancer with high-quality NGS, it was not possible to optimise the plasma collection time-points in this study. It is reasonable to hypothesise that transient rises in ctDNA may have occurred within the 72 h before venepuncture, and therefore were not detected. Acknowledging ctDNA’s half-life of up to 2 h, it is also possible that the processing times achieved (median 25 min to processing from collection, median 30 min further to freezing) affected the VAF values produced. However, EDTA tubes processed within 6 h have been found to have similar performance than cell-stabilising tubes for ctDNA analysis [42].

A key weakness of our study was the inability to differentiate between clonal haematopoiesis of indeterminate potential (CHIP) [43] and tumour-related mutations, due to the lack of tumour tissue or leukocytes available for NGS. *IDH2* and *TP53* genes, both identified in this study, are associated with CHIP. However, given the variation in levels observed over the short study period we believe that these mutations are unlikely to reflect to CHIP in these patients. Furthermore, the frequency of *TP53* mutations in this study (60%) is more in line with NSCLC (expected frequency of 60–70%) than with CHIP (expected frequency 2–5%).

Further investigation is required to address the many questions surrounding the interaction of radiotherapy with ctDNA kinetics if the full clinical utility of this technology-enabled assessment is to be realised. If ctDNA kinetics prove to have prognostic capability, surveillance strategies could be individualised for following each patient’s treatment. Early trends may be predictive for response to radiation therapy, and such a predictive biomarker could inform discussions with patients about dose-escalation/acceleration and addition of concurrent drugs. Whether the low dose bath effect of VMAT radiotherapy affects total cfDNA is not known and investigation of this is warranted. It is worth noting that cfDNA increases reduce VAF of ctDNA, given that this is a proportion, although this was not measured in our small sample set. Future investigations of ctDNA kinetics during radical radiotherapy should involve large patient cohorts powered to ensure the interpretation of their results can be justified.

In the oligometastatic setting, transient elevations in ctDNA following local consolidative radiotherapy could provide opportunity for understanding the uncontrolled tumour cell clones. In this way, a radiation-primed liquid biopsy would enable a non-invasive method of understanding mechanisms of resistance. Similarly, ongoing exploration of tumour evolution in lung cancer by the TRACERx group may create further opportunities for application of ctDNA in the future [44].

In summary this was a prospective observational pilot study of ultra-deep NGS ctDNA analysis in a cohort of stage II-III NSCLC undergoing the first week of state-of-the-art curative-intent radiotherapy. The feasibility of ctDNA analysis was shown in this small patient cohort with high-quality NGS and a larger study with a range of dose-fractionations, time-points and disease stages, including matched tissue analysis, will help addressing the clinical relevance of ctDNA monitoring during radiotherapy.

## Conclusion

This pilot study of ultra-deep NGS ctDNA analysis in non-metastatic NSCLC in the first week of radical radiotherapy demonstrated the feasibility of this approach. All included patients had detectable ctDNA at baseline, and had reduced levels at 7 days. A non-significant transient ctDNA increase at 72 h preceded the decrease observed at 7 days in 2 patients, in keeping with trends in the other limited data. Such temporary increases may represent a surge of cellular lethality very early (< 1 week) in courses of radiotherapy.

## Abbreviations

ctDNA: Circulating tumour DNA
NSCLC: Non-small cell lung cancer
CT/PET: Computed tomography/positron emission tomography
NGS: Next generation sequencing
cfDNA: Cell-free DNA
ECOG PS: Eastern Cooperative Oncology Group Performance Status
TNM8: Tumour-Node-Metastasis 8th Edition
MV: Megavoltage
EDTA: Ethylenediaminetetraacetic acid
SNV: Single nucleotide variant
CNV: Copy number variant
REC: Research Ethics Committee
VAF: Variant allele frequency
ASTRO: American Society for Radiation Oncology
